# Novel Antibiotic Resistance Genes Identified by Functional Gene Library Screening in *Stenotrophomonas maltophilia* and *Chryseobacterium* spp. Bacteria of Soil Origin

**DOI:** 10.3390/ijms24076037

**Published:** 2023-03-23

**Authors:** Laurita Klimkaitė, Ignas Ragaišis, Renatas Krasauskas, Modestas Ružauskas, Edita Sužiedėlienė, Julija Armalytė

**Affiliations:** 1 Institute of Biosciences, Life Sciences Center, Vilnius University, 10257 Vilnius, Lithuania; laurita.klimkaite@kc.vu.lt (L.K.); ignasraga@gmail.com (I.R.); renatas.krasauskas@gf.vu.lt (R.K.); edita.suziedeliene@cr.vu.lt (E.S.); 2 Microbiology and Virology Institute, Lithuanian University of Health Sciences, 44307 Kaunas, Lithuania; modestas.ruzauskas@lsmuni.lt

**Keywords:** *Stenotrophomonas maltophilia*, *Chryseobacterium* spp., functional gene library, antibiotic resistance genes, antibiotic resistance in soil, metallo-β-lactamase

## Abstract

As one of the most diverse habitats of microorganisms, soil has been recognised as a reservoir of both antibiotics and the antibiotic resistance genes (ARGs). Bacteria naturally inhabiting soil or water often possess innate ARGs to counteract the chemical compounds produced by competitors living in the same environment. When such bacteria are able to cause infections in immunocompromised patients, their strong innate antibiotic resistance mechanisms make treatment difficult. We generated functional gene libraries using antibiotic-resistant *Stenotrophomonas maltophilia* and *Chryseobacterium* spp. bacteria isolated from agricultural soils in Lithuania to select for the genetic determinants responsible for their resistance. We were able to find novel variants of aminoglycoside and β-lactam resistance genes, with β-lactamases isolated from the *Chryseobacterium* spp. functional gene library, one of which is a variant of IND-like metallo-β-lactamase (MBL) IND-17 and the other of which is a previously uncharacterised MBL we named CHM (*Chryseobacterium* metallo β-lactamase). Our results indicate that soil microorganisms possess a diversity of ARG variants, which could potentially be transferred to the clinical setting.

## 1. Introduction

As the most diverse habitat of microorganisms, soil has been recognised as a reservoir of both antibiotics and antibiotic resistance genes (ARGs) [1,2]. Naturally occurring ARGs have been observed to spread to clinical settings [3,4], contributing to the antibiotic resistance crisis. Additionally, novel ARGs can proceed to evolve in soil due to the human-derived influx of antibiotics discharged from health facilities or used for treatment of farm animals. Those compounds can reach the environment through sewage and animal manure and persist there [5]. Even low concentrations of antibiotics can possibly trigger specific responses in environmental bacteria and push them towards the generation of novel ARGs [6,7]. 

Opportunistic pathogens of environmental origin can be considered as another vector for the rapid advance of ARGs from soil to the clinical environment. Bacteria naturally inhabiting soil or water often possess innate ARGs to counteract the chemical compounds produced by competitors living in the same environment. When such bacteria cause infections in immunocompromised patients, their strong innate antibiotic resistance mechanisms make treatment difficult. Bacteria of genus *Stenotrophomonas* (phylum *Proteobacteria*) are universally found in the environment (soil, water, plants, animals, and sewage). *S. maltophilia* is considered the most widespread species and is known to cause infections in humans and animals as an opportunistic pathogen [8,9]. Another environmental bacterium, *Chryseobacterium* sp. (Phylum *Bacteroidetes*), which is found primarily in soil and water, has increasingly been found to colonize immunocompromised patients through contaminated medical devices and liquids [10,11]. Both bacteria have high innate antibiotic resistance, which complicates treatment of their infections. [9,11,12]. *S. maltophilia* possesses a wide array of innate resistance mechanisms, which include multidrug efflux pumps, enzyme-modifying β-lactam or aminoglycoside antibiotics, and reduced permeability of outer structures [9,13,14]. The resistance mechanism of *Chryseobacterium* spp. has been observed to involve β-lactamases and possibly efflux mechanisms [15,16]. As the ARGs utilised by opportunistic pathogens of environmental origin often differ from the ARGs typically encountered in the clinical environment, their mechanisms and potential threat should be explored. 

We recently selected antibiotic-resistant bacteria from agricultural soils in Lithuania [17] and observed that the isolates of *Stenotrophomonas* spp. and *Chryseobacterium* spp. were among the most antibiotic-resistant bacteria selected. We then generated functional gene libraries to identify the novel genetic determinants responsible for the innate resistance. 

## 2. Results

### 2.1. The Selection and Characterisation of Resistant S. maltophilia and Chryseobacterium spp. from the Soil

The bacteria were isolated from three farming fields in Lithuania during the year 2016 [17]. The sample collection points represented two different types of farming, i.e., organic and conventional (intensive); in all cases, the type of the soil was sandy loam. The organic farming sites were known not to use inorganic fertilizers or pesticides for a time period of over 20 years and were fertilized only with organic fertilizers (farmyard manure and slurry). Identification of bacterial isolates was based on 16S rRNA fragment sequencing as described previously [18]. All six isolates of *S. maltophilia* were collected from a conventional winter wheat farming field. Antibiotic-resistant *Chryseobacterium* spp. isolates were recovered from various fields (conventional winter wheat, conventional rapeseed, and organic rapeseed). Two of the isolates were identified as *C. aahli*, and another two were identified as *C. soldanellicola*; for four of the isolates, only the genus could be assigned as *Chryseobacterium* spp. All selected isolates of *S. maltophilia* and *Chryseobacterium* spp. were resistant to at least two classes of antibiotics (β-lactams, aminoglycosides, and/or fluoroquinolones) (Table S1). 

### 2.2. Functional Gene Library Construction and Evaluation

To search for novel ARGs, total DNA was extracted from six *S. maltophilia* and eight *Chryseobacterium* spp. isolates, fragmented, and cloned to pBluescript KS (-) plasmid. Several *Escherichia coli* libraries were created, and their general features are described in Table 1. The probability of full genome coverage was calculated according to Seidman (2010) [19], and for 99% probability of full coverage, 2.4 × 10^4^ clones were needed to represent the analysed *S. maltophilia* genomes, and 1.4 × 10^4^ clones were needed for *Chryseobacterium* spp. genomes. The constructed libraries achieved a full genome coverage for the representation of both *S. maltophilia* and *Chryseobacterium* spp. isolates.

### 2.3. Selection of Resistant Clones

*E. coli* BL-21 (DE3) containing a plasmid library were plated on LB containing antibiotics to select resistant clones. The number of library clones was calculated to exceed the number of unique clones in the library by at least 50 fold (Table 1 and Table 2). The concentration of antibiotics used for the selection was at least two times higher than the minimal inhibitory concentration (MIC) values of the *E. coli* containing an empty pBluescript KS (-) vector. The antibiotics used for selection were aminoglycosides (gentamycin, kanamycin, and streptomycin), β-lactams (cephalosporin cefuroxime and carbapenem imipenem), tetracycline, chloramphenicol, and ciprofloxacin. The isolated clones exhibiting at least a four-fold MIC increase compared to *E. coli* containing an empty pBluescript KS (-) vector were selected for further analysis.

### 2.4. Identification of ARGs from S. maltophilia Functional Gene Library

The screening of the functional gene library of *S. maltophilia* revealed only aminoglycoside-resistant clones. All the clones found to be resistant to kanamycin were carrying the same gene responsible for the resistance, which was a putative APH(3′) family aminoglycoside O-phosphotransferase, as inferred by protein homology. The highest protein identity (99%, Protein BLAST) was to APH(3′)-II family aminoglycoside O-phosphotransferase from *S. maltophilia* (NCBI Reference Sequence WP_100465153.1). Cloning to the pET-218 expression vector and induction of gene expression caused kanamycin resistance (Table 3) but not gentamycin resistance, which is consistent with the APH(3′)-II family [20,21]. Although the gene sequence was only 73% identical to the closest homologue, which was previously confirmed to be involved in the antibiotic resistance of *S. maltophilia* [20], the location on the chromosome was similar. Therefore, the gene should be annotated as *aph(3′)-IIc* and is a variant of the previously described aminoglycoside resistance gene.

All streptomycin-resistant clones carried a gene encoding a putative APH(6) family aminoglycoside O-phosphotransferase. The closest protein homologue (72%) was APH(6) family putative aminoglycoside O-phosphotransferase from *Stenotrophomonas* spp. HMWF003 (GenBank: PTT58433.1). Cloned into an expression vector putative APG(6) gene conferred resistance to streptomycin (Table S3) but not to other aminoglycosides, consistent with the other APH(6) variants [21]. The closest protein homologues with confirmed resistance were the aminoglycoside phosphotransferases located in Tn5 of *Klebsiella pneumoniae* (identity 48%, UniProt: P13082) and other *Enterobacteria* [22]. However, the immediate neighbourhood of the *aph(6)* gene of *S. maltophilia* was not similar to a transposon or any other possibly mobile elements. 

Both APH(3′)- and APH(6)-encoding genes were detected in a majority (86 and 87%, respectively) of *Stenotrophomonas* spp. genomes sequenced and assembled to date, with APH(3′) more conserved (58–90% identity) than APH(6) (64–73% identity). To assess the representation of the genes in the genomes of *S. maltophilia* isolates used for gene library construction, the primers for the detection of the genes were then created (Table S2) (positioned on the most conserved parts of the genes, as distinguished by multiple alignments of the available homologues). Both genes were detected by PCR in four out of six *S. maltophilia* isolates originally used for library construction; the other two strains had either one gene or the other. 

### 2.5. Identification of ARGs from the Chryseobacterium spp. Functional Gene Library

The screening of the *Chryseobacterium* spp. functional gene library revealed clones resistant to several antibiotic classes (Table 2). Streptomycin resistance was due to the aminoglycoside 6-adenylyltransferase (ANT(6)) family protein-encoding gene, which is widely spread among *Chryseobacterium* genus bacteria, sharing high homology (62–93% identity), and was detected in 65% of *Chryseobacterium* spp. genomes sequenced and assembled to date. The gene was found in the *Chryseobacterium* spp. genomes of both environmental and clinical origin, as well as in other *Bacteroidetes* (orders *Sphingobacteriales* and *Flavobacteriales* (*Sphingobacterium*, *Pedobacter*, *Flavobacterium*, *Elizabethkingia*, and *Myroides*). 

Tetracycline-resistant clones detected from the *Chryseobacterium* spp. gene library all carried the tetracycline resistance MFS efflux pump gene. Analysis showed that all sequenced and assembled genomes of *Chryseobacterium* spp. possessed the MFS efflux pump gene, and almost one-third of genomes had several variants of this gene. Protein BLAST revealed similar MFS transporters present in other environmental *Bacteroidetes* of orders *Sphingobacteriales* and *Flavobacteriales* (*Sphingobacterium*, *Pedobacter*, *Flavobacterium*, *Elizabethkingia*, and *Myroides*). 

Two distinct *Chryseobacterium* spp. genes were found to confer resistance to β-lactam antibiotics. One of them, IND-like MBL (IND-17) protein, was selected twice during both imipenem and cefuroxime screening (Table 2). When cloned into an inducible pET-28b vector and induced, the gene conferred resistance to tested cephalosporins and carbapenems (Table 3). The protein sequence was closest to the predicted IND family subclass B1 MBL from *Chryseobacterium* spp. T16E-39 (90% identity) (NCBI reference sequence: WP_089026551.1). Among the IND-like MBLs from *Chryseobacterium* spp. previously demonstrated to be functional, the closest homologue (78% identity) was IND-4 (GenBank: AAG29765) from *Chryseobacterium indolgenes*. We also noticed the genetic neighbourhood of the IND-17-encoding gene was very similar to other IND-like MBL genes in *Chryseobacterium* spp., as previously observed [15]. Furthermore, 48% of *Chryseobacterium* spp. genomes that have been sequenced and assembled to date had a version of the IND-like MBL gene (77–100% identity), all located in similar positions in the chromosome. 

**Figure 1:**
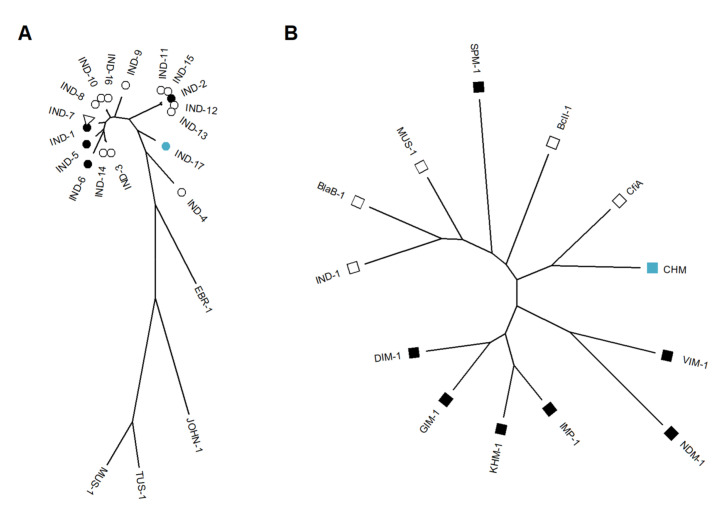
Phylogenetic trees of the identified MBLs. (**A**) Unrooted tree of typical *Flavobacteriaceae* MBLs [26], including all IND MBL variants. Full circles represent MBLs with determined kinetic properties, empty circles represent variants lacking kinetic data, triangles indicate determined enzyme structure, and the newly identified IND variant (IND-17) is represented by a full blue circle. (**B**) Unrooted tree of most notable B1 subclass MBLs. Full squares represent plasmid-associated MBLs, empty squares represent chromosome-associated MBLs [27], and the newly identified MBL CHM is identified by a blue square.

IND-17, along with IND-4, are more distant from other IND variants described to date (Figure 1A). Common structural features of B-1 subclass MBLs are Loop 1 and Loop 2, which are formed by amino acids in positions 60–64 and 221–241, respectively; amino acid residues are numbered according to the BBL numbering scheme for the class B β-lactamases [23]. These loops are thought to be responsible for substrate binding. In IND-7 Loop 1 was demonstrated to be formed by VFGGK residues [24], which are well conserved in all IND variants. Only IND-17 has a substitution of K64R of all IND variants, while Loop 2 is less conserved, and IND-like MBL does not have unique amino acid substitutions (Figure S2) [25]. Zn ion-binding amino acids are conserved in all B-1 subclass MBLs.

When selecting the clones on cefuroxime, another β-lactamase CHM (from *Chryseobacterium* metallo β-lactamase)-encoding gene was identified. When cloned to an inducible vector, the gene was able to confer resistance to cephalosporins and carbapenems (Table 3). The protein was similar to subclass B1 MBLs from *Chryseobacterium* spp. OV705 (NCBI reference sequence: WP_047494176.1) with 99% identity and contained a CcrA-like MBL-B1 domain. Although according to the closest homologue, the protein could be assigned to B1 MBL subclass, it had a very low similarity (26% identity to IND-like MBL described above and 24% identity to IND-1 (Genbank: AF099139)) to previously described IND-like MBLs, which are considered to be the genus-specific β-lactamases of *Chryseobacterium* spp. [15]. The gene was found in 51% of *Chryseobacterium* spp. chromosomes sequenced and assembled to date and was located in a different region than IND-like MBL. The protein also differed from another *Chryseobacterium* spp. B3 subclass MBL, CPS-1 (NCBI reference sequence: WP_063857696.1) (identity of only 14%), which was previously described in [28]. Using the IMG database [29] 44 fully sequenced *Chryseobacterium* genomes were found, 21 of which had CHM homologues that were not IND. A total of 17 of those genomes were isolated in a clinical setting, 3 were associated with plants, and 1 was from an animal sample. The origin of 23 genomes lacking CHM were equally distributed among clinical (8), environmental (7), and animal-associated (8) samples.

The newly identified MBL CHM is more related to CfiA (CcrA) than to other relevant B-1 subclass β-lactamases and groups with chromosome-associated MBLs (Figure 1B). A class B β-lactamase phylogenetic study recently grouped previously uncharacterised MBL gene families [30]. The CHM groups with Gene Family 16 identified by constructing a maximum-likelihood phylogenetic tree and should be considered as subclass B1.3 β-lactamase, and their genes are typically found in the *Bacteroidetes* phylum (Figure S3). This MBL does not have closely characterised homologues to determine structural or functional features of this protein, but amino acids binding Zn1 (His116, His118, and His196) and Zn2 (Asp120, Cys221, and His263) are conserved as in all B-1 subclass MBLs.

### 2.6. Purification and Kinetic Parameters of IND-17 and CHM β-Lactamases 

For IND-17 and CHM kinetic activity evaluation, proteins without a signal peptide were purified using ion-exchange chromatography. CHM was purified to >85% purity, and IND-17 was purified to >95% purity as observed in protein analysis by gel electrophoresis (Figure S1). 

IND-17 was able to hydrolyse ampicillin, cephalosporins, and carbapenems (Table 4). β-lactams were hydrolysed with similar catalytic efficiency (10^−6^–10^−7^ s^−1^ µM^−1^), while cephalosporins had the least efficient hydrolysis and ceftazidime was not hydrolysed. Compared to IND-6 and other MBLs, IND-17 has poor kinetic activity, which might be a result of amino acid substitution in Loop 1. CHM was able to hydrolyse cephalosporins and carbapenems but not ampicillin and had a higher kinetic activity spectrum than IND-17, with ceftazidime catalytic efficiency being the lowest (10^−8^ s^−1^ µM^−1^), while the catalytic efficiency of cefuroxime was the highest (>10^−6^ s^−1^ µM^−1^). While CHM showed average catalytic efficiency in hydrolysing imipenem, it had relatively high efficiency in hydrolysing meropenem. 

IND-17 and CHM did not hydrolyse ceftazidime and ampicillin, respectively, consistently with MIC results.

## 3. Discussion

*Stenotrophomonas maltophilia* and *Chryseobacterium* spp. are widespread environmental bacteria mostly found in soil and water sources, while in the clinical setting they are known as multidrug-resistant opportunistic human pathogens causing serious infections in immunocompromised patients [8,32,33,34]. Resistance to different classes of antibiotics is known to be the key trait allowing opportunistic pathogens to survive in hospitals and cause life-threatening infections [35,36]. It has been proposed that some clinical infections might be caused by bacteria not associated with the hospital environment but by isolates living naturally in the wild [37]. Therefore, the resistance mechanisms of environmental bacteria and underlying ARGs could also be transferred to clinical settings and exchanged with other pathogens.

*Stenotrophomonas maltophilia* is known to be resistant to different classes of antibiotics [38,39]. Antibiotic-modifying enzymes, efflux pumps, and reduced membrane permeability represent the main resistance mechanisms of *S. maltophilia* conferring resistance to β-lactam antibiotics (including cephalosporins and carbapenems), macrolides, aminoglycosides, chloramphenicol, tetracyclines, and polymyxins [8,40]. Comparative analysis of ARGs in environmental and clinical *S. maltophilia* isolates have shown similar ARG profiles in both groups [41,42]. The environmental *S. maltophilia* isolates analysed in this study were also resistant to multiple antibiotics, including aminoglycosides, β-lactams, and fluoroquinolones (Table S1). Using a functional gene library approach, we were able to identify *S. maltophilia* ARGs *APH(3′)-II* and *aph(6)* genes responsible for resistance to aminoglycoside kanamycin and streptomycin, respectively. We also expected to find other ARGs determining resistance to β-lactams and ciprofloxacin; however, we were unable to select clones bearing resistance to either cefuroxime or imipenem, nor to ciprofloxacin. Several *S. maltophilia* β-lactamases have been described previously [43], with chromosome-encoded β-lactamases *blaL1* and *blaL2* being studied the most. *blaL1* MBL is known to hydrolyse penicillins, cephalosporins, and carbapenems, and *blaL2* serine-β-lactamase is known to hydrolyse penicillins, cephalosporins, and aztreonam [44,45,46]. They were also shown to be widespread in *S. maltophilia* of clinical origin [47]. We were able to detect them using a PCR approach in one and four strains (out of six *S. maltophilia* strains selected for gene bank construction), respectively. The inability of a functional gene library to select for *blaL1* or *blaL2* could indicate complex regulation of gene expression, as previously observed [48], as they could not confer carbapenem resistance to the *E. coli* host. Interestingly, even the isolates without detected *blaL1* and *blaL2* genes showed resistance to imipenem, confirming that the mechanisms of *S. maltophilia* antibiotic resistance are still unclear [49]. Unexpectedly, we also did not detect any of the efflux pumps during the screening of the library, even though we recently showed that the same *S. maltophilia* isolates exhibited efflux-mediated resistance [17]. Inability of the functional gene library to select for the efflux genes could be due to the size of the library inserts or the incompatibility with the library host *E. coli*. 

Although genus *Chryseobacterium* is composed of more than 130 species [50], the majority of information about *Chryseobacterium* antibiotic resistance has been obtained from three main human-infecting species: *C. indologenes*, *C. meningosepticum*, and *C. gleum* [51]. Clinical *Chryseobacterium* isolates are known to be highly resistant to most β-lactam agents, aminoglycosides, tetracyclines, macrolides, chloramphenicol, erythromycin, and ticarcillin-clavulanate [11,26,52,53]. Information about the resistance of the environmental *Chryseobacterium* spp. is limited to several studies [33,54,55]. A recent study by Mwanza et al. (2022) showed that out of 38 environmental and clinical *Chryseobacterium* species tested, *C. gleum*, *C. indologenes*, *C. joostei*, *C. daecheongense*, *C. daeguense*, *C. shigense*, *C. soldanellicola*, *C. soli*, *C. ureilyticum*, *C. vrystaatense*, and *C. wanjuense* were resistant to most of the tested antimicrobials [33]. We identified genes conferring resistance to tetracycline, streptomycin, β-lactams, cefuroxime, and imipenem from environmental *Chryseobacterium* spp. Resistance to tetracycline was caused by the MFS efflux pump gene. Tetracycline efflux resistance genes *tetA* and *tetD* are known to be present in genus *Chryseobacterium* [56,57], and efflux has been suggested as a mechanism of *Chryseobacterium* spp. resistance [16,58,59]. However, no specific *Chryseobacterium* proteins responsible for tetracycline resistance have been confirmed yet. *Chryseobacterium* resistance to aminoglycosides is also well documented [11]; efflux pumps and antibiotic-modifying enzymes have been shown to be coded in sequenced *Chryseobacterium* genomes [53,60]. In this study, we found *Chryseobacterium* streptomycin resistance gene *ant(6)*, which is widespread in genus *Chryseobacterium*. Interestingly, other *ant(6)* variants are mostly present in Gram-positive bacteria and are often associated with transposons and plasmids [21,61]. The closest gene neighbourhood of the putative *Chryseobacterium* spp. *ant(6)* did not contain any apparent transposons or other possibly mobile elements. 

*Chryseobacterium* resistance to β-lactams is known to be caused by several β-lactamases. IND β-lactamases are the most abundant and well known for antibiotic resistance in clinical settings and were shown to successfully hydrolyse penicillins, cephalosporins, and carbapenems [26]. Two extended-spectrum β-lactamases (ESBL) of Ambler class A have been found in clinical strains: CIA from *C. indologenes* [62] and CGA from *C. gelum* [63]. Subclass B3 ESBL CPS β-lactamase was found in bacteria from soil [28]. In this study, we identified two novel *Chryseobacterium* spp. MBLs: IND-17 and CHM. IND-17 is a novel IND variant that hydrolyses a broad spectrum of β-lactams (penicillins, cephalosporins, and carbapenems) but does not display high catalytic constants. Lys64 substitution to Argin predicted Loop 1 structure could influence the enzymatic activity, as it is considered a part of the active site of β-lactamases. CHM is a B1.3 subclass MBL that is capable of hydrolysing cephalosporins and carbapenems but not ampicillin. Almost all B1.3 subclass MBLs have been detected in the *Bacteroidetes* phylum, only four of which (ORR, ECV, MYO, and ZOG) have been experimentally described [64]. None of these MBLs have been kinetically characterised. Some putative gene groups of B1.3 MBLs were found to be specific to *Chryseobacterium* spp., including Gene Family 16, to which CHM belongs. Although sequences of these genes can be found in databases (GenBank, etc.), there are no data attributing them as typical *Chryseobacterium* MBLs. As genes of CHM are usually found in *Chryseobacterium* genomes, and majority of clinical strains possess its homologues; therefore, CHM can be considered as a newly described typical *Chryseobacterium* MBL. 

In conclusion, during the screening of functional gene libraries of two soil bacteria for the novel ARGs, we were able to find novel variants of aminoglycoside and β-lactam resistance genes. Although the *S. maltophilia* isolates used for library construction possessed species-specific β-lactamases, our screening only revealed aminoglycoside phosphotransferase genes, indicating complex regulation of *S. maltophilia* β-lactamase expression. Two β-lactamases were isolated from the *Chryseobacterium* spp. functional gene library, one of which is a variant of IND-like MBL, which we named IND-17, and the other of which is a previously uncharacterised MBL, which we named CHM. All the selected genes were predicted to be located on bacterial chromosomes and where not identical to their previously known clinically relevant homologues, indicating their origins as soil organisms. Our results indicate that soil microorganisms that moonlight as opportunistic pathogens possess a diversity of ARG variants and are a possible source of novel ARGs transferred to clinical settings. 

## 4. Materials and Methods

### 4.1. The Bacteria and Plasmids Used in This Study 

Six *Stenotrophomonas maltophilia* and eight *Chryseobacterium* spp. antibiotic-resistant isolates were used for genomic library construction; all bacteria were isolated from farming field soil in Lithuania during the year 2016 [17,65]. The isolates were designated as resistant if the MIC of antibiotic value matched EUCAST clinical breakpoints (v. 7.0, 2017, PK/PD (non-species related). Species were identified by matching obtained sequences with a sequence showing the highest maximum identity score from the GenBank database. If the identity of the best match was <99% and the query cover was <96%, only the genus was assigned. All environmental isolates were grown in LB medium at 28 °C.

All *Escherichia coli* strains and plasmids used in this study are described in Table S3. Strains were grown in LB medium (supplemented with antibiotics ampicillin or kanamycin for plasmid selection if needed) at 37 °C unless otherwise indicated. 

### 4.2. Genomic Library Preparation and Characterisation 

Genomic DNA from selected *Stenotrophomonas maltophilia* and *Chryseobacterium* spp. isolates were extracted using a GeneJET genomic DNA purification kit (Thermo Fisher Scientific, Vilnius, Lithuania). The resulting DNA was then fragmented with Sau3AI (Thermo Fisher Scientific) under non-optimal empirically determined conditions to obtain DNA fragments with sizes around 1 kb–3 kb. The fragments were purified using electroelution from agarose gel, followed by ethanol precipitation. The purified DNA was ligated into BamHI-digested pBluescript KS(-) plasmid for 16 h at +22 °C at a plasmid:insert the ratio of 2:1 (*w*/*w*). Ligate was then transformed into electrocompetent *E. coli* BL21 (DE3) cells that were prepared as described by Warren (2011) [66] with the following modifications: O/N-grown *E. coli* BL21(DE3) was diluted 100× into 200 mL of fresh in SOB medium and grown at +37 °C with agitation to an OD600 of 0.4–0.6. The culture was chilled on ice for 15 min and harvested by centrifugation at 1000× *g* for 5 min at +4 °C. The bacteria were washed twice with ice-cold water by centrifugation at 1000× *g* for 6 min at +4 °C, followed by a final wash with filter-sterilized 10% mannitol in water (*w*/*v*) at 1000× *g* for 7 min at +4 °C. The prepared cells were gently resuspended in the remaining solution after decantation. *E. coli* JM107 were prepared as described, except all washing procedures were performed using water. Then, 70 μL of fresh electrocompetent cells was used for transformation of the 1 μL ligation reaction in a 1 mm wide cuvette using 1800 V. Transformed cells were recovered using 1 mL of SOC medium for 1.5 h with shaking, followed by plating onto LB agar plates with ampicillin at 100 μg/mL and subsequent incubation at +30 °C for 24 h. The transformants were collected by adding 3 mL of PBS into each plate and scraping all colonies with a plate spreader. The pooled fractions were mixed, aliquoted, centrifuged at 5000× *g* for 10 min at +4 °C, decanted, and stored at −80 °C. 

The average lengths of insert of libraries were evaluated as follows: plasmids from 17 random transformants were extracted as described in [67], except phenol extraction of DNA was performed before isopropanol precipitation. The extracted DNA was then digested with HindIII, and the resulting fragment lengths were analysed by comparison with the pBluescript KS(-) profile using agarose gel electrophoresis. The full library size was approximated by multiplying the average insert size by the number of collected colonies. The obtained number was compared to the theoretical value provided by Clarke and Carbon [68], which defines the minimum amount of clones needed to isolate an individual sequence from a library with the given probability. 

### 4.3. Selection and Characterisation of Resistant Library Clones 

For resistant *Stenotrophomonas maltophilia* and *Chryseobacterium* spp. libraries clone selection pooled fractions of transformants were spread on LB medium containing the antibiotic of interest (10 mg/L gentamicin, 20 mg/L kanamycin, 6 mg/L tetracycline, 40 mg/L, 80 mg/L streptomycin, 16 mg/L chloramphenicol, 0.5 mg/L ciprofloxacin, 16 mg/L cefuroxime, or 0.5 mg/L imipenem) and incubated overnight at 37 °C. The diversity of clones was assessed by resistant clone plasmid hydrolysation with PvuII restriction endonuclease, and unique plasmids were selected for further analysis. For elimination of spontaneous resistant mutants, unique plasmids were retransformed to *E. coli* BL-21 (DE3), MIC values were determined. Plasmids conferring 4 or more times MIC difference compared to the control strain (*E. coli* BL-21 (DE3) with pBluescript without insert) were sequenced and aligned to the closest DNA sequences available in GenBank using BLASTN. The region was then analysed for annotated genes related to antibiotic resistance. Oligonucleotide primers were created, and the predicted ARGs were cloned to either pET-28b or pET-218 to confirm the resistance phenotype of the gene (Table S2). Another set of internal primers was used to detect the presence of the gene of interest in all the remaining non-identical clone groups (Table S2). 

### 4.4. Determination of Minimal Inhibitory Concentration (MIC)

MIC assays were performed using the broth dilution method as described previously [69] and evaluated according EUCAST clinical breakpoints (v. 11.0, 2021). Briefly, overnight cultures of *E. coli* BL-21 (DE3) containing plasmids of interest (at a final concentration of 5 × 10^5^ CFU/mL) were inoculated into sterile round-bottom 96-well plates containing LB medium supplemented with 1 mM IPTG and 2-fold dilutions of analysed antibiotics. Bacterial growth was evaluated after incubation at 37 °C for 24 h.

### 4.5. Plasmid Construction 

All reagents for cloning were obtained from Thermo Fisher Scientific, and procedures were performed according to the manufacturer’s recommendations. pET-218 was obtained by cloning polylinker from pET-28a into pET-21d via XbaI-XhoI. For functional evaluation of putative resistance genes, as well as for IND-17 and CHM proteins (without signal peptide) purification, DNA sequences were amplified from original library plasmids using high-fidelity Phusion polymerase (primers are listed in Table S2) and cloned into pET-28b or pET-218 plasmids hydrolysed with NcoI restriction endonuclease and blunted with T4 polymerase. *E. coli* JM107 strain was used for the transformation required for cloning. All final constructs were confirmed by Sanger sequencing.

### 4.6. Purification of CHM and IND-17 β-Lactamases

CHM and IND-17 β-lactamases were purified from *E. coli* BL21Tuner (DE3) cultures containing pET-28b plasmids with cloned respective genes. Overnight cultures grown at 37 °C were diluted 100-fold into fresh media containing 60 mg/L kanamycin and grown until OD_600_ = 0.5–0.6. The grown cultures were prechilled on ice for 15 min before supplementing the media with a 0.1 mM final IPTG concentration. The cultures were then transferred to a bacterial shaker chilled to 16 °C and left to grow for 16 h with shaking. Grown cells were collected by centrifugation at 5000× *g* for 10 min at 4 °C, resuspended in a column-binding buffer (50 mM potassium acetate, pH 5.25 (for CHM), and 50 mM sodium phosphate, pH 7.4 (for IND-17)) containing 1 mM PMSF, and lysed using ultrasound. Cell lysates were clarified by centrifugation at 12,000× *g* for 30 min at 4 °C and filtered through 0.22 μm PVDF syringe filters (Carl Roth). The CHM from the lysate was purified by first loading onto a 1 mL HiTrap SP Sepharose Fast Flow column (Cytiva) equilibrated with 50 mM potassium acetate at pH 5.25. The protein was eluted with 0 to 0.5 M K_2_SO_4_ gradient in the same buffer. The collected fractions containing the enzyme were polled and dialysed into 20 mM Tris-H_2_SO_4_ (pH 9.0) using a 5 mL HiTrap desalting column (Cytiva). MBL was then loaded onto a 1 mL HiTrap Q Sepharose XL column equilibrated with 20 mM Tris-H_2_SO_4_ (pH 9.0) and eluted using 0 to 0.5 M K_2_SO_4_ gradient in the same buffer. The fractions with the purified protein were pooled and dialysed into 50 mM sodium phosphate buffer (pH 7.4) using a 5 mL HiTrap desalting column (Cytiva).

IND-17 β-lactamase was purified by loading the lysate onto a 1 mL HiTrap SP Sepharose Fast Flow column (Cytiva) equilibrated with 50 mM sodium phosphate (pH 7.4) and eluted with 0 to 0.5 M K_2_SO_4_ gradient in the same buffer. The collected fractions containing the enzyme were polled and filtered through an Amicon Ultra-15 50K centrifugal filter device. The flowthrough was then loaded onto an Amicon Ultra-15 10K centrifugal filter device, concentrated, and dialysed into 50 mM sodium phosphate buffer (pH 7.4) according to the manufacturer’s recommendations. The purified proteins were aliquoted and stored at −80 °C. The protein concentration was determined using a ROTI^®^Nanoquant Bradford assay according to the manufacturer’s recommendations. Protein purity was determined from 12% SDS-PAGE.

### 4.7. CHM and IND-17 β-Lactamases Kinetic Activity Evaluation 

Kinetic measurements were performed at 22 °C in 50 mM HEPES buffer (pH 7.0) supplemented with 50 µM ZnCl_2_ in a reaction volume of 200 µL. Rates of antibiotic hydrolysis were measured using a GENESYS 10S UV-Vis spectrophotometer (Thermo Scientific). The kinetic parameters were determined by measuring the initial hydrolysis rate at various antibiotic concentrations as described previously [70]. Extinction coefficients and measuring wavelengths for antibiotics were used: nitrocefin (ε_486_ = 20,500 M^−1^ cm^−1^), ampicillin (ε_235_ = −1560 M^−1^ cm^−1^), cefazolin (ε_260_ = −1560 M^−1^ cm^−1^), cefuroxime (ε_260_ = −9500 M^−1^ cm^−1^), ceftazidime (ε_260_ = −13900 M^−1^ cm^−1^), imipenem (ε_295_ = −6800 M^−1^ cm^−1^), meropenem (ε_300_ = −10000 M^−1^ cm^−1^).

### 4.8. Bioinformatic Analysis 

The closest gene or protein homologues were determined using the NCBI BLAST tool. MEGA 11 software was used for protein sequence alignment using MUSCLE with default parameters, phylogenetic tree construction using default neighbour-joining parameters, and visualisation. The NCBI GenBank accession numbers for proteins used in phylogenetic trees and sequence alignments are: IND-1–AAD20273, IND-2–AAG29757, IND-3–AAG29761, IND-4–AAG29765, IND-5–AAS78754, IND-6–CAJ32373, IND-7–ABO21412, IND-8–ACZ65152, IND-9–ACZ65153, IND-10–ADA13241, IND-11–ADK25050, IND-12–ADK25051, IND-13–AAG29760, IND-14–ADK38716, IND-15–BAJ14288, IND-16–ALP75901, EBR-1–AAN32638, JOHN-1–AAK38324, MUS-1–AAN63647, TUS-1–EKB08120, BlaB-1–AAF89154, BcII-1–AAA22276, CfiA–AAA22907, SPM-1–AAR15341, VIM-1–CAC35170, NDM-1–AHM26723, IMP-1–ABK27309, KHM-1–BAF91108, GIM-1–ALO69078, and DIM-1–AGC92784. 

To determine the prevalence of the ARGs in the sequenced genomes, TBLASTN was used against the fully assembled genomes of *Stenotrophomonas* sp. (111 genomes) or *Chryseobacerium* sp. (79 genomes) (https://www.ncbi.nlm.nih.gov/assembly, date of retrieval 22 February 2023). The hits with an E value <0.001, coverage > 50%, and identity > 50% were selected as homologues, and their prevalence (%) was calculated.

## Figures and Tables

**Table 1:** The characterisation of constructed functional gene libraries of *S. maltophilia* and *Chryseobacterium* spp.

Library	Number of Clones	Average Insert Size (kb ± Standard Deviation)	Total DNA (Mb)
*S. maltophilia*			
S_malt_pBLU_2	9.6 × 10^4^	3.7 ± 1.5	355
*Chryseobacterium* spp.			
C_sp_pBLU_1	1.1 × 10^4^	4.8 ± 2.2	53
C_sp_pBLU_2	1.4 × 10^4^	4.2 ± 0.6	59

**Table 2:** *S. maltophilia* and *Chryseobacterium* spp. ARGs identified by functional gene library screening.

Selection on Antibiotic (mg/L)	Clones Tested	No. of Resistant Clones	No. of Unique Resistant Groups	No. of Clones with Resistance Gene	Resistance Gene Detected	GenBank Accession Number
*S. maltophilia* gene library S_malt_pBLU_2/*E. coli* BL21(DE3)						
Kanamycin (20)	1.2 × 10^6^	98	3	98 *	*aph(3′)-IIc* aminoglycoside O-phosphotransferase gene	MK374278
Streptomycin (40)	1.2 × 10^6^	37	3	37 *	Putative *aph(6)* family aminoglycoside O-phosphotransferase gene	MK374279
*Chryseobacterium* spp. gene libraries C_sp_pBLU_1, C_sp_pBLU_2/*E. coli* BL21(DE3)						
Streptomycin (80)	1.7 × 10^6^	181	1	181	Putative *ant(6)* aminoglycoside adenylyltransferase family gene	MK401903
Tetracycline (6)	1.4 × 10^6^	90	2	90 *	Tetracycline resistance MFS efflux pump gene	MK401905
Cefuroxime (16)	7.4 × 10^5^	127	2	125	IND-like metallo-β-lactamase gene	MK401904
				2	Putative metallo-β-lactamase gene	MK401906
Imipenem (0.5)	3.0 × 10^6^	17	1	17	IND-like metallo-β-lactamase gene	MK401904

* Several groups of unique inserts were detected among the resistant clones; however, they all contained the same gene, as confirmed by PCR.

**Table 3:** Antibiotic resistance of ARGs from *S. maltophilia* and *Chryseobacterium* spp. ARGs were cloned to IPTG-inducible pET vectors (pET-218 for aminoglycoside resistance genes and pET-28b for β-lactam resistance genes), and MICs were tested under inducing conditions (1 mM IPTG).

Gene Origin	ARG	Originally Isolated on Antibiotic	Ampicillin	Cefazolin	Cefuroxime	Ceftazidime	Ceftriaxone	Imipenem	Meropenem	Streptomycin	Kanamycin	Gentamicin
*S. maltophilia*	*APH(3′)-II*	Kanamycin	-	-	-	-	-	-	-	6	100	3
*S. maltophilia*	*aph(6)*	Streptomycin	-	-	-	-	-	-	-	100	3	3
*Chryseobacterium* spp.	IND-17 gene	Imipenem/ cefuroxime	>3200	>64	>256	5	>64	>256	>256	-	-	-
*Chryseobacterium* spp.	CHM gene	Cefuroxime	12	>64	>256	160	>64	64	>256	-	-	-

- not determined.

**Table 4:** Hydrolysis of antibiotics by IND-17 β lactamase and CHM. K_M_ is presented in µM, k_cat_ in s^−1^, and k_cat_/K_M_ in s^−1^ µM^−1^. Mean values with standard errors are shown for K_M_.

	IND-17			CHM			IND-6 ^a^	NDM-1 ^b^	IMP-1 ^b^	VIM-1 ^b^
Antibiotic	K_M_	k_cat_	k_cat_/K_M_	K_M_	k_cat_	k_cat_/K_M_	k_cat_/K_M_	k_cat_/K_M_	k_cat_/K_M_	k_cat_/K_M_
Nitrocefin	152 ± 4.7	68	0.44	153 ± 10	74	0.48	2.67	-	-	-
Ampicillin	470 ± 108	148	0.31	NH	NH	NH	-	0.66	0.48	-
Cefazolin	38 ± 12	5.0	0.13	167 ± 27	126	0.75	-	-	-	-
Cefuroxime	30 ± 5.8	8.3	0.27	76 ± 19	124	1.63	3.62	0.61	0.22	0.55
Ceftazidime	NH	NH	NH	200 ± 41	3.9	0.02	0.27	0.03	0.18	0.9
Imipenem	185 ± 19	48	0.26	389 ± 48	78	0.20	37	0.21	1.2	0.99
Meropenem	258 ± 25	66	0.25	10 ± 2.0	9.8	0.96	0.17	0.25	0.12	0.28

NH—no hydrolysis; -—data not available; ^a^—From Zeba et al., 2009 [26]; ^b^—From Yong et al., 2009 [31].

## Data Availability

The data presented in this study are openly available. The gene sequences were submitted to GenBank under accession numbers MK374278, MK374279, MK401903, MK401905, MK401904, MK401906, and MK401904.

## Supplementary Materials

The following supporting information can be downloaded at: https://www.mdpi.com/article/10.3390/ijms24076037/s1.
