# Supplementary material for: Novel Antibiotic Resistance Genes Identified by Functional Gene Library Screening in Stenotrophomonas maltophilia and Chryseobacterium spp. Bacteria of Soil Origin
Source: Int J Mol Sci. 2023 Mar 23;24(7):6037. doi: 10.3390/ijms24076037 (PMC10094639; doi:10.3390/ijms24076037)
Supplement: Supplementary file 1 [file ijms-24-06037-s001.zip › Supp Figure S1 Proteins purification.pdf]

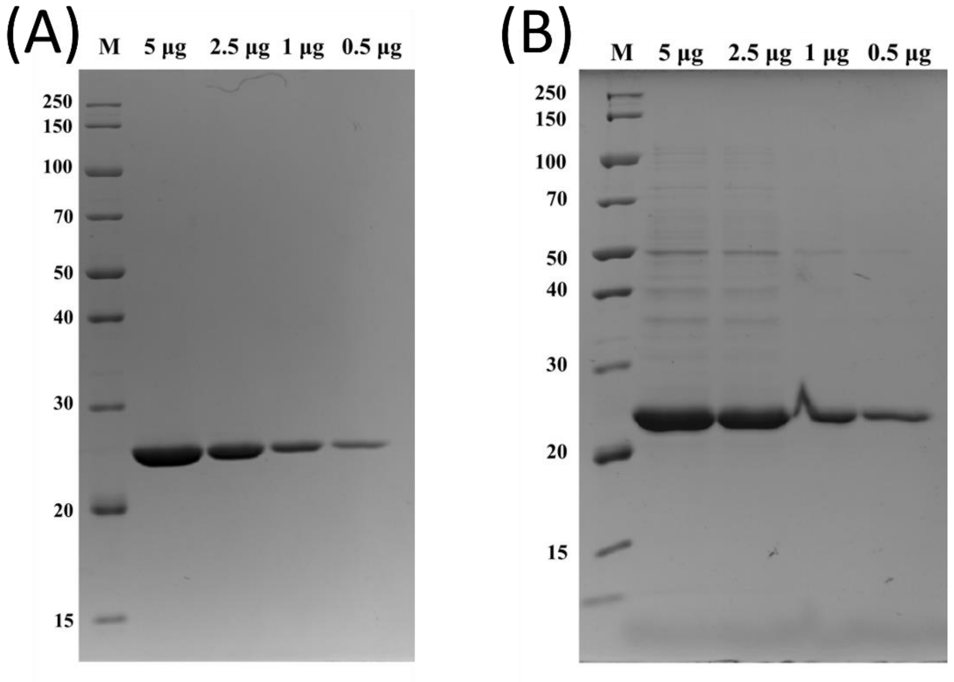

Figure S1. SDS-polyacrylamide gel of purified IND-like MBL and MBL β-lactamases from *Chryseobacterium* sp. genomic libraries. (A) IND-17 MBL and (B) CHM β-lactamases purified using Ion exchange chromatography. M - PageRuler™ Unstained Broad Range Protein Ladder (Thermo Scientific™), 5 µg, 2.5 µg, 1 µg and 0.5 µg – purified proteins. IND-17 was purified to >95% purity, CHM was purified to >85% purity as judged from 5 µg protein in gel electrophoresis.
