# Supplementary material for: Novel Antibiotic Resistance Genes Identified by Functional Gene Library Screening in Stenotrophomonas maltophilia and Chryseobacterium spp. Bacteria of Soil Origin
Source: Int J Mol Sci. 2023 Mar 23;24(7):6037. doi: 10.3390/ijms24076037 (PMC10094639; doi:10.3390/ijms24076037)
Supplement: Supplementary file 1 [file ijms-24-06037-s001.zip › Supp Figure S2 IND alignment.pdf]

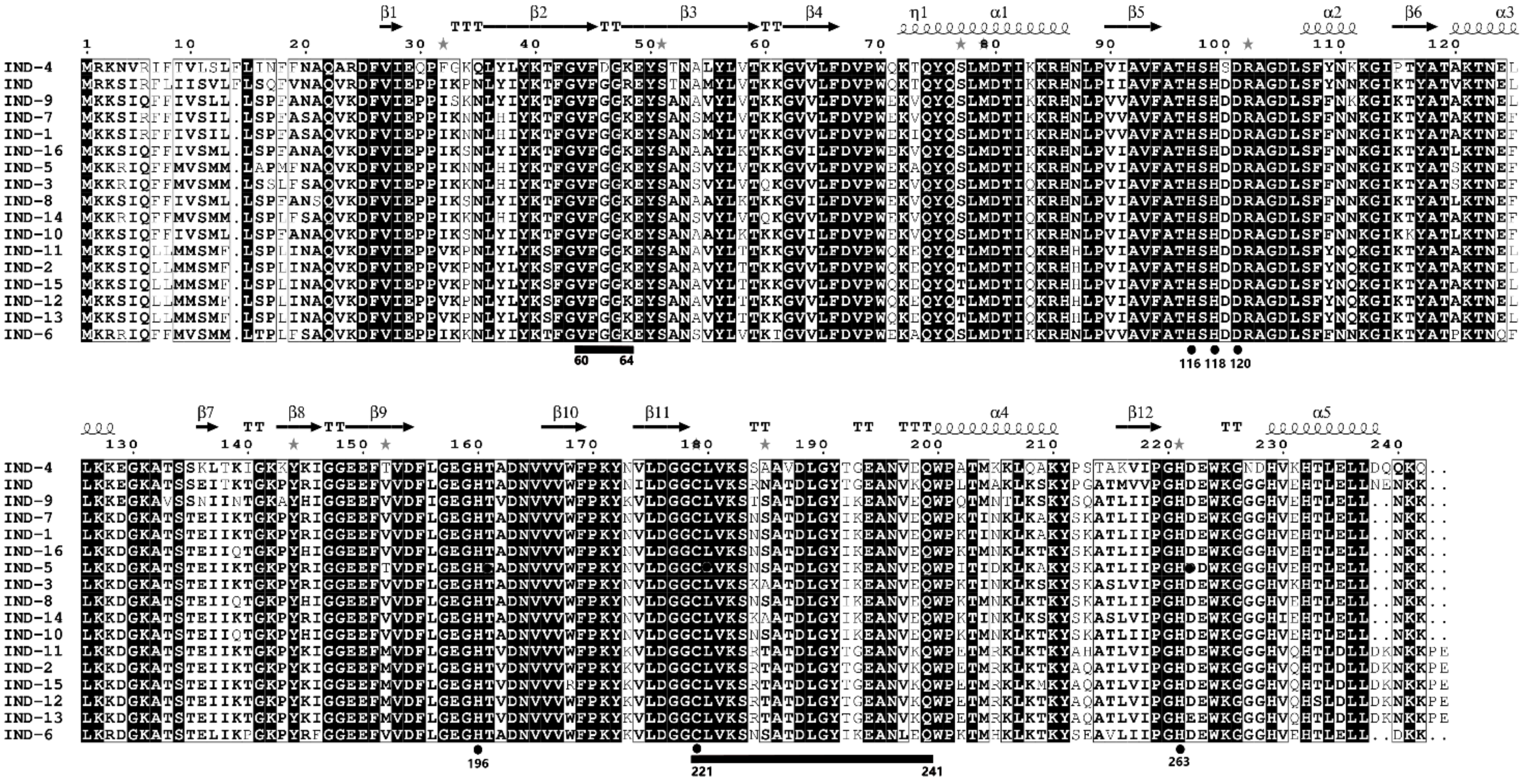


**Figure S2**. Multiple sequence alignment of IND all known IND variants, visualised using ESPript 3.0 online tool [25]. Newly found IND-17 is named IND. Numbers on top of alignment represent amino acid sequence number from N-terminus. BBL numbering of amino acids is presented below the alignment. Lines represent Loop 1 at 60-64 and Loop 2 at 221-241. Dots under alignment mark Zn ions binding amino acids.
