# Supplementary material for: Novel Antibiotic Resistance Genes Identified by Functional Gene Library Screening in Stenotrophomonas maltophilia and Chryseobacterium spp. Bacteria of Soil Origin
Source: Int J Mol Sci. 2023 Mar 23;24(7):6037. doi: 10.3390/ijms24076037 (PMC10094639; doi:10.3390/ijms24076037)
Supplement: Supplementary file 1 [file ijms-24-06037-s001.zip › Supp Figure S3 MBL Phylogenetic Tree.pdf]

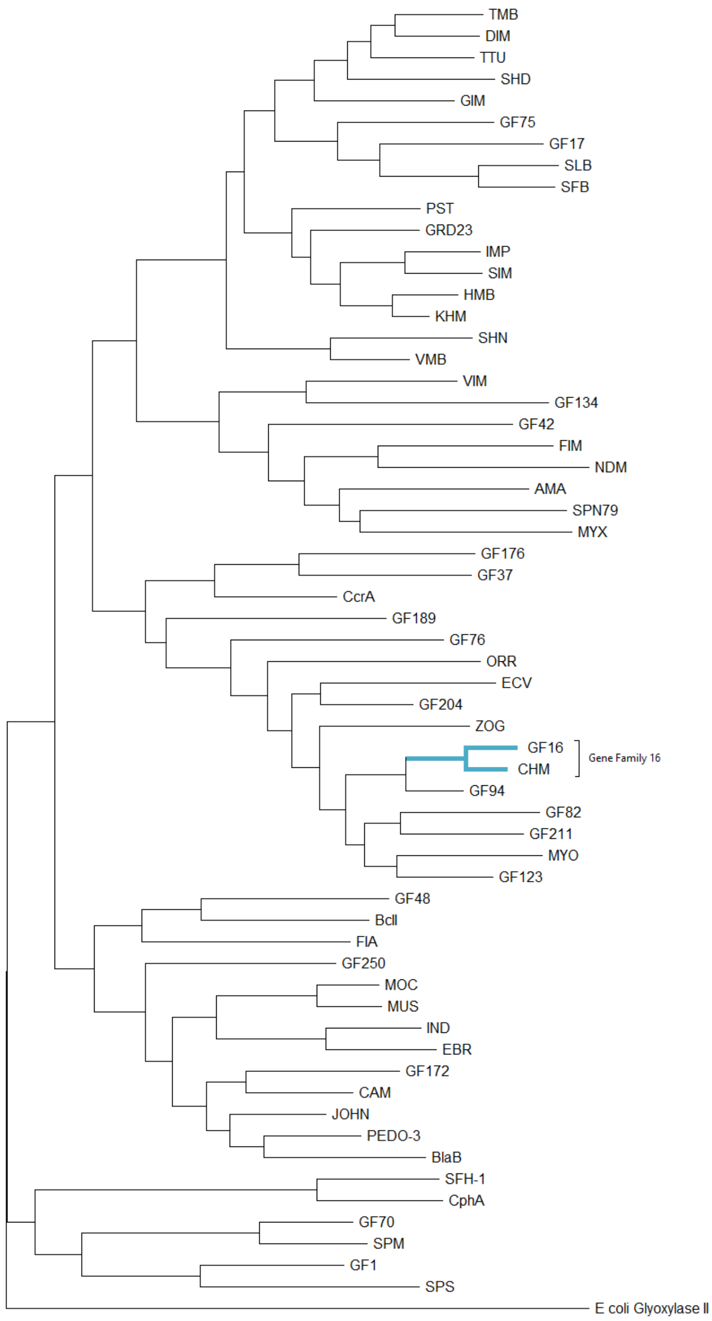

Figure S3. Phylogenetic tree of B1 subclass β-lactamases using protein sequences adapted from Berglund et al., 2020 publication [30]. Named MBL sequences were used from Beta Lactamase Data Base, using first variant of β-lactamases presented in database. GF (Gene Family) sequences were used from Berglund et al., 2020 [30] supplementary data, using first sequence from each Gene Family.
