# Supplementary material for: Novel Antibiotic Resistance Genes Identified by Functional Gene Library Screening in Stenotrophomonas maltophilia and Chryseobacterium spp. Bacteria of Soil Origin
Source: Int J Mol Sci. 2023 Mar 23;24(7):6037. doi: 10.3390/ijms24076037 (PMC10094639; doi:10.3390/ijms24076037)
Supplement: Supplementary file 1 [file ijms-24-06037-s001.zip › Supp Table S2 Primers.pdf]

Table S2. Primers used in the study.

Primer	Sequence 5‘ - 3‘	Purpose of the primer
2S_APH(6)_clon_F<br>2S_APH(6)_clon_R	AGCGAACCCTACCTGAGCCG<br>TCACTTCTCCGCCAGCGCCT	Cloning of *S. maltophilia* APH(6) family gene
2S_APH(3)_clon_F<br>2S_APH(3)_clon_R	GGAGCACCCGATCCCTTCA<br>TCAGAAGAACTCGTCGAGCA	Cloning of *S. maltophilia* APH(3') family gene
2C_aden_trans_clon_F<br>2C_aden_trans_clon_R	AAAATAAGAGAAGATAAGCTTGA<br>ACACATCAGGAATTCTGGTTG	Cloning of *Chryseobacterium* sp. ANT(6) family gene
2C_TETR_MFS_clon_F<br>2C_TETR_MFS_clon_R	GAAAATTCAAGGAAAAAAGCTG<br>CTATTTTTTAATATCCTTCTTTTTT	Cloning of *Chryseobacterium* sp. tetracycline MFS efflux pump gene
2C_IND_clon_F<br>2C_IND_clon_R	AGAAAAAGTATTCGATTTTTAATTA<br>TAGCCTCGAGCTATTTTTTATTTTCA TTTAGAAGT	Cloning of *Chryseobacterium* sp. IND-like MBL gene
2C_Met_beta_lact_clon_F<br>2C_Met_beta_lact_clon_R	AAAACTACCCTTACCCATATAG<br>TAGCCTCGAGTTATTGAGTTTTAAA TAATGTAATG	Cloning of *Chryseobacterium* sp. putative MBL gene
Steno_kanAPH(3)_F<br>Steno_kanAPH(3)_R	CSTTCSTGAARTCGGAAGTGATCG<br>CCRCARTCRATGAAACCRCTGAA	Detection of *S. maltophilia* APH(3') family gene
Steno_strepAPH(6)_F<br>Steno_strepAPH(6)_R	NCCRCASAGRTCCGGRTTG<br>GGYGACCTGCAYCAYGACAA	Detection of *S. maltophilia* APH(6) family gene
Chrbct_Ind_F<br>Chrbct_Ind_R	GCAACACATTCGCATGATGATA<br>TGKCCRCCKCCTTYCCATT	Detection of *Chryseobacterium* sp. IND-like MBL gene
Chrbct_StrR_F<br>Chrbct_StrR_R	TCCYYATGCWCCKGTAGATGA<br>TCCCACCARAAATCATTNAKC	Detection of *Chryseobacterium* sp. ANT(6) family gene
Chrbct_TetrMFS_F<br>Chrbct_TetrMFS_R	GYTDGGWTTTGCSTAYGC<br>RCRAACATCATCCADCCYT	Detection of *Chryseobacterium* sp. tetracycline MFS efflux pump gene
blaL1_Sm_F<br>blaL1_Sm_R	ACCACACCTGGCAGATCGG<br>TCGCCATCCATGATGATGCG	Detection of *S. maltophilia blaL1*
blaL2_Sm_F<br>blaL2_Sm_R	GAGCGCTTCCCGATGTGCA<br>CATTGCTGCCGGTCTTGTC	Detection of *S. maltophilia blaL2*
IND_sig_F<br>IND_R	CAGGTAAGAGATTTTGTGATC<br>CTATTTTTTATTTTCATTTAGAAGT	IND-like MBL purification
MBL_sig_F<br>MBL_R	TGTGGCTCTCAACATAAAG<br>TTATTGAGTTTTAAATAATGTAATG	Putative MBL purification
