# Supplementary material for: Novel Antibiotic Resistance Genes Identified by Functional Gene Library Screening in Stenotrophomonas maltophilia and Chryseobacterium spp. Bacteria of Soil Origin
Source: Int J Mol Sci. 2023 Mar 23;24(7):6037. doi: 10.3390/ijms24076037 (PMC10094639; doi:10.3390/ijms24076037)
Supplement: Supplementary file 1 [file ijms-24-06037-s001.zip › Supp Table S3 strains and plasmids.pdf]

Table S3. Strains and plasmids used in this study

Strains/plasmids	Description	Reference
**Strains**		
E.coli BL-21(DE3)	*F*$^{-}$ *ompT gal dcm lon hsdS*$_{B}$(*r*$_{B}^{-}$*m*$_{B}^{-}$) *λ(DE3 [lacI lacUV5-T7p07 ind1 sam7 nin5]) [malB*$^{+}$*]*$_{K\text{-}12}$(*λ*^{S})	Studier and Moffatt, 1986
E. coli Tuner (DE3)	F^{-} *ompT hsdS*$_{B}$ (r$_{B}^{-}$ m$_{B}^{-}$) *gal dcm lacY1*(DE3)	Novagen
E.coli JM107	*endA1 glnV44 thi-1 relA1 gyrA96 Δ(lac-proAB) [F′ traD36 proAB*$^{+}$ *lacI*^{q} *lacZΔM15] hsdR17(R*$_{K}^{-}$ *m*$_{K}^{+}$) *λ*$^{-}$	Yanisch-Perron, C., Vieira, J., and Messing, J. (1985) Gene 33, 103.
pBluescript_KS(-)	Cloning vector	Novagen
**Plasmids**		
pET-28b	Protein expression vector (kanamycin resistance)	Novagen
pET-218	Protein expression vector (ampicilin resistance)	This work
pET28_TETR_MFS	*Tetr_MFS* from *Chryseobacterium* spp. genomic library cloned to pET-28b	This work
pET28_IND	*IND-17* gene from *Chryseobacterium* spp. genomic library cloned to pET-28b	This work
pET28_MBL	*CHM* gene from *Chryseobacterium* spp. genomic library cloned to pET-28b	This work
pET21_APH(3)	*aph(3′)-II* gene from *Stenotrophomonas maltophilia* library cloned to pET-218	This work
pET21_APH(6)	*aph(6)* gene from *Stenotrophomonas maltophilia* library cloned to pET-218	This work
pET28_IND-sign	*IND-17* gene without signal peptide sequence cloned to pET-28b	This work
pET28_MBL-sign	*CHM* gene without signal peptide sequence cloned to pET-28b	This work
